# Phytoestrogens by inhibiting the non-classical oestrogen receptor, overcome the adverse effect of bisphenol A on hFOB 1.19 cells

**DOI:** 10.22038/ijbms.2020.45296.10545

**Published:** 2020-09

**Authors:** Zar Chi Thent, Gabriele Ruth Anisah Froemming, Aletza Binti Mohd Ismail, Syed Baharom Syed Ahmad Fuad, Suhaila Muid

**Affiliations:** 1 Faculty of Medicine, Universiti Teknologi MARA, Sungai Buloh Campus, 47000 Selangor, Malaysia; 2 Faculty of Medicine and Health Sciences, Universiti Malaysia Sarawak (UNIMAS), 94300 Kota Samarahan, Sarawak, Malaysia; 3 Institute for Pathology, Laboratory and Forensic Medicine (IPPerForM), Universiti Teknologi MARA, Sungai Buloh Campus, 47000 Selangor, Malaysia

**Keywords:** Bisphenol A, ERRG, hFOB 1.19 cells, Oestrogens receptors, Phytoestrogens

## Abstract

**Objective(s):**

Since bisphenol A (BPA) induces bone loss and phytoestrogens enhance the osteoblastogenesis by binding to the non-classical and classical oestrogen receptors, respectively, the present study was aimed to observe the osteoprotective effect of phytoestrogens on BPA-induced osteoblasts in hFOB 1.19 cells.

**Materials and Methods:**

All groups of hFOB 1.19 cells were induced with 12.5 μg/ml of BPA except the control (Ctrl) group. Meanwhile, treated groups received phytoestrogens; Daidzein (Dz), Genistein (Gt), Equol (Eq) and 17β-oestradiol (Est) in different concentrations for 24 hr duration.

**Results:**

We found that the protein expression of non-classical oestrogen-related receptor (ERRG) was highly expressed in BPA group, whereas classical oestrogen receptor alpha (ERα) and oestrogen receptor beta (ERβ) were relatively increased with phytoestrogens treatment under BPA exposure. The dense actin cytoskeletal filaments were also observed. qRT-PCR showed up-regulation of mitogen-activated protein kinase 3 (MAPK3) and G protein-coupled receptor 30 (GPR30) expressions; significant down-regulation of ERRG and up-regulation of ERα and ERβ were observed in phytoestrogens-treated cells, which was supported by the increased expressions of oestrogen receptor 1 (ESR1) and oestrogen receptor 2 (ESR2).

**Conclusion:**

Phytoestrogens improved the deteriorative effect of BPA via down-regulation of ERRG in hFOB 1.19 cells. This study showed that the efficacy of consumption of phytoestrogens in rendering them as potential therapeutic strategy in combating the adverse bone effects of BPA.

## Introduction

Oestrogen has a key role in bone remodelling process by exerting effects on cell-cell communication and shape, which coordinate cells. Oestrogen deficiency can disturb the cellular metabolism, which leads to developing osteoporosis by significantly reducing the bone mass (1). It is believed that bisphenol A (BPA) mimics the oestrogen action; however, it interacts differently with the ligand binding domain of the oestrogen receptor and recruits different transcriptional co-regulators. The industrial chemical like BPA used in the production of plastic products can bind to the non-classical oestrogen receptor like oestrogen-related receptor gamma (ERRG) as well as to the G-protein coupled receptor 30 (GPR30) and, therefore, activate oestrogen-sensitive genes via the non-genomic pathways (2). BPA binds with high affinity to ERRG, which is a nuclear receptor with unclear physiological ligand (3). BPA is a weak endocrine disruptor (EDC) due to its 1000–10,000 fold lower binding capacity to the classical oestrogen receptors α and β (ESRα & β) compared to 17β-oestradiol (Est) (4). Inhibition of ERRG expression significantly increased bone morphogenetic protein 2 (BMP2)-induced osteoblast differentiation, osteocalcin expression, alkaline phosphatase (ALP) activity and bone mineralization (5). 

The alterations in cell function such as changes in cell-cell communication, cell structure, gene expression, cell metabolism and cell function are mainly controlled by the oestrogenic hormones. Hormonal modulations affect the physical components of a cell that consist of nucleus, cytoskeleton and the extracellular matrix. All these components orchestrate cellular events necessary for gene expression that preserve the cell specificity and tissue phenotypic properties (6). High affinity binding of BPA to ERRG depletes the oestrogen, which results in reduction in the cellular pool of osteoblast and an increase in differentiation of osteoclast progenitors. Oestrogen replacement therapy has been an effective strategy for prevention and treatment of bone loss (7). Due to the undesirable side effects of hormonal replacement therapy, phytoestrogens, the naturally occurring selective oestrogen receptor modulators, have been recommended as alternatives for the treatment of bone loss and osteoporosis (8). Among the phytoestrogens, isoflavones are shown to enhance the bone mineralization in ovariectomized animals and postmenopausal women (9, 10). Daidzein, a major isoflavone found in soybean, exerts oestrogenic action by binding to the oestrogen receptors (11, 12). It regulates the expressions of interleukin-6, osteoprotegerin, and receptor activator of nuclear factor kappa B (NF-κB) ligand in the human foetal osteoblast (hFOB1.19) cell line (13). On the other hand, Equol, a metabolite of daidzein, was shown to inhibit bone loss in ovariectomized animals through activating the oestrogen receptor (14). Findings of an *in vitro* study showed that equol inhibited the osteoclastogenesis induced by vitamin D supplement (15). Genistein is a non-steroidal phyto-oestrogen that controls the osteoblast metabolism through oestrogen receptor -dependent pathways (16). Although several studies have highlighted on the oestrogenic effect of phytoestrogens towards bone loss, there is paucity of research investigated the effect of each phytoestrogen on BPA-induced osteoblasts cells under oestrogen pathway and thus, awaits further clarification.

We aimed to study the bone-sparing effect of phytoestrogens through ER-dependent signal pathways. We investigated the effects of each phytoestrogen on hFOB 1.19 cells and the possible signal pathways of ESR α, ESRβ, and mitogen-activated protein kinase kinase/extracellular regulated kinase (MAPK/ERK). Taken together, our data explained that phytoestrogens overcome bone loss effect of BPA via up-regulation of classical oestrogen receptor and down-regulation of non-classical oestrogen receptor and showed the potential value of phytoestrogens as a nutraceutical agent for clinical application in BPA-related bone loss. 

## Materials and Methods

***Materials***

Human foetal osteoblasts 1.19 (hFOB 1.19) cells were purchased from Addexbio, USA. Cell culture reagents- Dulbecco’s Modified Eagle Medium: Nutrient Mixture F-12 (DMEM/F12), penicillin, streptomycin and foetal bovine serum were sourced from Biosera Laboratories, France. Ascorbic acid and β-glycerophosphate were purchased from Sigma, St. Louis, MO, USA. Bisphenol A (BPA), Daidzein (Dz), Genistein (Gt), Equol (Eq) and 17-β oestradiol (Est) were sourced from Abcam (Cambridge, UK). Enzyme linked immunosorbent assay (ELISA) kits for the protein expression of oestrogen receptor β (ERβ), oestrogen receptor α (ERα) and ERRG were purchased from Fine Biotech, China. Total protein extraction kit was purchased from Bio-Rad, USA, and actin staining from CellLight® Reagent *BacMam 2.0, ThermoFisher Scientific. All other chemicals used were sourced from Institute of Medical Molecular Biotechnology (IMMB), Universiti Teknologi MARA (UiTM).

***Cell culture and sub-culturing***

In the current study, hFOB 1.19 cells, passage 4, were used. These immortalized cells are ideal for the expression of all the specific molecular markers and undergo well differentiation when grown in the medium containing ascorbic acid and β glycerolphosphate for 6 days. The subculturing was performed by growing active hFOB 1.19 cells in a growth medium consisting of DMEM F-12 supplemented with 10% fetal bovine serum (FBS) and 1% penicillin/streptomycin (antibiotic/antimycotic). The cells were incubated in 95% air and 5% CO_2_ at 37 ^°^C until 80% confluence was reached. Before commencing the experiments, cultured cells were fed twice a week. The adhered cells were then released from the flask using cells detachment solution (Accutase). Following 6 days of differentiation period, the treatment was started.

***Cell culture treatment***

The cells were counted using a hemocytometer and were seeded at a density of 1×10^4 ^cells/cm^2^ in 96-well plates. Prior to the treatment, a stock solution (50 mg/ml) of BPA, Dz, Gt and Est were prepared using differentiation medium. The entire prepared medium was sterilized using 0.2 mm syringe filter (Sartorius, Germany). hFOB 1.19 cells were induced with BPA and treated with phytoestrogens including Dz, Gt, Eq, and Est. Single concentration of BPA 12.5 μg/ml was used. All the groups were stimulated with BPA 12.5 µg/ml except the Control (Ctrl) group. Ctrl group only received differentiation medium. All the treated groups were compared with (untreated) BPA group. Three (3) non-toxic concentrations of each phytoestrogen were chosen following MTT assay; Dz (5, 10, 20); Gt (10, 20, 40); Eq (1, 2, 4) and Est (2, 4, 16) µg/ml were considered as treatment groups. The treatment was continued for 24 hr.

***Analysing the protein expression of oestrogen receptors***

In this study, the potential biological influence of phytoestrogens including Dz, Gt, and Eq on the BPA-induced hFOB 1.19 cells was observed by analysing the sequential expression and regulation of human oestrogen receptors such as ERβ, ERα and oestrogen-related receptor gamma (ERRG) by using commercially available kits. These proteins are major phenotypic markers for osteoblast formation. Prior to the investigation, the cells (1×10^6^) were seeded in T-25 culture flasks. Following the differentiation period, cells were treated with different concentrations of Dz, Gt, Eq and Est in the presence of BPA except in the Ctrl group. Following 24 hr of incubation period, the cultured cells underwent total protein extraction by using ReadyPrep protein extraction kit. Then, the protein expression of each oestrogen receptors was estimated using sandwich ELISA. We followed the manufacturer’s instruction and at the end of investigations, colour changes from yellow to blue were noted. The OD value was recorded at 450 nm with a microplate reader (Brand, Country) and the colour intensity represented the expression concentration of each protein marker in the sample. 

***Cytoskeleton (Actin) staining***

Both the treated and untreated cultured adherent cells were seeded at the density of 1×10^5 ^in the 6-well plate. For the actin staining, the total percentage of cell confluent 70% was used following 24 hr of incubation period. The integrity and the morphology of actin were examined with phalloidin staining. Then, the appropriate volume of CellLight reagent for the number of cells was calculated. 

A CellLight® reagent for hFOB 1.19 cells about 20 particles per cell (PPC) was used following optimization. With gentle mixing, the calculated volume of CellLight^®^ reagents was directly added to the cells in the complete medium and mixed gently. Then, the cells were incubated overnight at 37 ^°^C. Following the incubation, the cells were viewed under inverted fluorescence microscope (Meiji Techno MT6000 Epi-Fluorescence Microscope). 

***Gene expression analysis by quantitative real time PCR (qRT-PCR)***

Cells were harvested at an amount of 1×10^7^ in cryovials, and then 350 μl of RNeasy Lysis Buffer (RLT) buffer was added in the cell pellet. Then, total RNA was obtained using All prep RNA extraction kit from Qiagen (Germany). Total RNA quality was measured, and RNA concentration was determined using the Nanodrop 2000 Spectrophotometer (Thermo Fisher, USA). Approximately 400 ng of total RNA was reverse transcribed to cDNA by using qPCRBIO cDNA synthesis kit (PCR biosystem, UK). Then, 1 μl of cDNA was mixed with SYBR Green Master Mix from PCR biosystem (UK) and primers for target and reference genes (Table 1 shows the list of genes and sequences). qPCR was performed using the CFX96 system from Bio-Rad (USA) and gene expression analysis was performed using CFX Manager^TM^ software (Bio-Rad, USA) with ^Δ^C_t_ of the sample normalized against reference genes (HPRT1 and ACTB1). 

***Statistical analysis***

In this study, data analysis was performed using one-way ANOVA followed by Bonferroni test by using SPSS version 22.0. All the experiments were performed in triplicate. Data were presented as mean±standard error of mean (SEM). A significant difference was considered *P<0.05.

## Results

**Figure 1:**
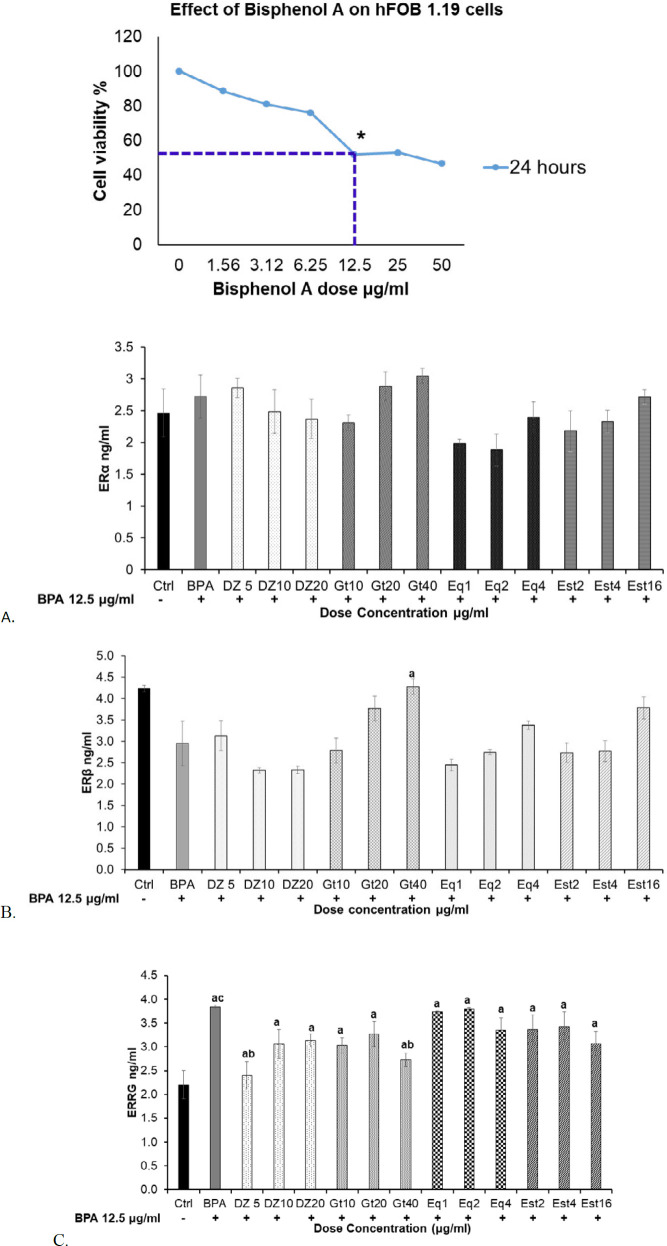
Protein expression of oestrogen receptors in hFOB 1.19 cells induced with BPA following treatment with Dz, Gt and Eq. (A) ERα, (B) ERβ and (C) ERRG. The expressions of treated groups were compared to the BPA group. The IC_50_ value of BPA on hFOB 1.19 cells was shown. Data were expressed as mean±SEM (n=9). In 1A and B, *P<0.05 was considered to be significant difference. In 1C, ^a^*P<*0.05 compared to BPA group, ^ab^*P<*0.01 compared between phytoestrogens groups, ^ac^*P<*0.05 compared to the Ctrl group

In our earlier study, we optimised the concentrations of BPA and phytoestrogens by using the hemocytometer. We observed cell viability rate in hFOB 1.19 cells stimulated with different concentrations of BPA for 24 hr and found that the 12.5 μg/ml concentration of BPA showed IC_50_ value (Figure 1). For the treatment groups, the three non-toxic concentrations of Dz (5,10,20); Gt (10,20,40); Eq (1,2,4) and Est (2,4, 16) µg/ml were chosen following the MTT assay (17). Treatments were given along with 12.5 μg/ml of BPA except in the Ctrl group. 

***Protein expression of oestrogen receptors***

The protein expression of phytoestrogens on BPA-stimulated hFOB 1.19 cells was analysed by using commercially available kit. The expression of classical oestrogen receptors including ERα, and ERβ and non-classical oestrogen receptor of ERRG were determined. Following 24 hr incubation period, the BPA group showed no significant difference in expression of ERα and ERβ (2.7±0.33 ng/ml and 3.0±0.51 ng/ml, respectively) when compared to Ctrl group (2.46±0.37 ng/ml and 4.2±0.08 ng/ml, respectively) (Figure 1A, B). While in the experimental groups treated with phytoestrogens, Dz, Gt and Eq, no significant changes were observed in expression of ERα and ERβ as compared to the BPA group (Figure 1A, B). However, Gt40 group showed significant increase in ERβ protein expression as compared to the other phytoestrogens groups (*P<*0.05). It was observed that both BPA and phytoestrogens had similar effects on classical oestrogen receptors. However, the insignificant up-regulation of both ERα and ERβ was noted in the phytoestrogen-treated groups (Figure 1A, B). To gain further insight into the difference between xenoestrogen (BPA) and phytoestrogens, their effect on ERRG was observed. The BPA group showed significant up-regulation (*P<*0.05) of ERRG level (3.8±0.04 ng/ml) when compared to the Ctrl group (2.2±0.29 ng/ml) (Figure 1C). However, phytoestrogens-treated cells showed down-regulation of ERRG level, particularly in Dz5 (2.4±0.28 ng/ml) and Gt40 (2.7±0.14 ng/ml) groups (*P<*0.05) compared to BPA group (Figure 1C). Treatment with Eq also showed down-regulation of ERRG level, though the effect was insignificant compared to the other phytoestrogen-treated cells. We also observed the oestrogenic potency of Est group and it showed no significance difference compared to the phytoestrogens-treated cells (Figure 1C). 

***Actin cytoskeletal structure***

**Figure 2.**
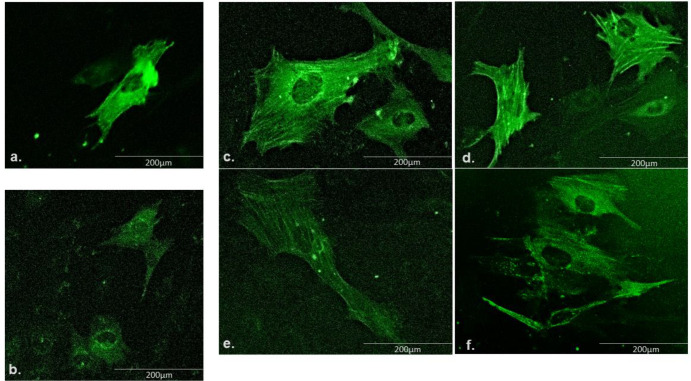
Immunofluorescence images of the CellLight phalloidin stained hFOB 1.19 cells. a) Ctrl b) BPA c) Dz5 d) Gt40 e) Eq4 and f) Est 16 μg/ml treated cells showing apparent difference in the density of f-actin filaments. All groups of cells were exposed with BPA 12.5 μg/ml except for the (a) Ctrl group

It has been reported that the filaments of actin cytoskeleton are important in forming a pre-stressed mechanical network that maintains the structure and stiffness of the cell (18). Therefore, it is crucial to observe the actin structures to understand the underlying reason for the changes in the osteoblasts elasticity. Fluorescence images of CellLight phalloidin-stained cells (Figure 2) showed that the actin filaments were less dense in the BPA group of cells as compared to the Ctrl group. BPA induced decrease in the density of actin filaments of hFOB 1.19 cells, which can result in decreasing the stiffness of the cells. Based on the findings from protein expression of ERRG, we assumed that 16 μg/ml of the Dz5, Gt 40, Eq4 and Est have potential osteogenic effect against BPA. Therefore, the cells from these treatment groups were selected to observe the density of actin filament under fluorescence microscope. Co-treatment of phytoestrogens with the BPA enhanced the density of actin filaments in osteoblast-like cells. Prominently increase in the density of actin filaments were found in Dz5 and Gt40 groups (Figure 2C, D), whereas Eq 4 group showed less prominent actin filaments (Figure 2E). Moreover, Est16 group was also observed to have increased actin filament density under BPA exposure (Figure 2F). 

***Regulation of phytoestrogens on BPA-induced gene expression***

*Genes for cell proliferation and differentiation*

**Figure 3:**
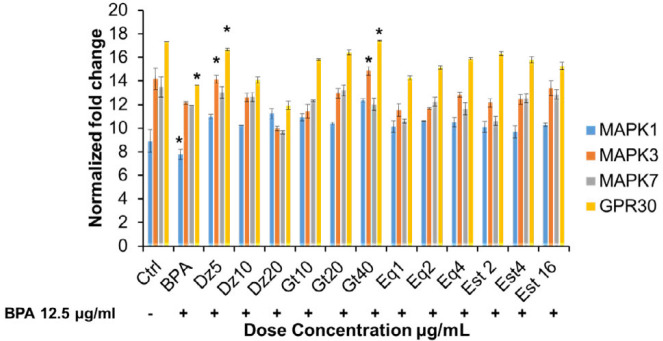
Gene expression of osteoblast proliferation and differentiation markers in BPA-induced phytoestrogen-treated hFOB 1.19 cells. Total RNA extracted from all groups of hFOB 1.19 cells was reverse transcribed and amplified with real-time PCR using specific primer sequences. Table 1: The gene expression was normalized against reference genes (HPRT1 and ACTB1) for all group of cells. Data are expressed as mean±SEM triplicate independent biological samples. *P<0.05; is considered significant

Using qRT-PCR, we found that BPA dramatically reduced MAPK1, MAPK3, MAPK7 and GPR30 over 24 hr period. Each gene displayed a different sequential temporal pattern of gene induction (Figure 3). MAPK1, MAPK3, MAPK7 and GPR30 are associated with osteoblast proliferation and differentiation. The expression level in BPA alone group was compared with Ctrl group under normalized fold change. The BPA group showed down-regulation of MAPK1, MAPK3, MAPK7 and GPR30 expression compared to Ctrl group (Figure 3). However, significantly (*P*<0.05) decreased expressions of MAPK1 and GPR30 were noticed in BPA group compared to low concentration of Dz5 and high concentration of Gt40 μg/ml groups. For the treated groups, we found that phytoestrogens as well as Est groups did not show any significant difference in expressions of MAPK1 and MAPK7 levels compared to the BPA group. Interestingly, the significant (*P<*0.05) upregulated expression of MAPK3 and GPR30 were observed in Dz5 and Gt40 μg/ml groups when compared among the treatment groups (Figure 3). Est groups showed relatively increased expressions of MAPK3 and GPR30. 

*Gene expression of the oestrogen receptors*

**Figure 4:**
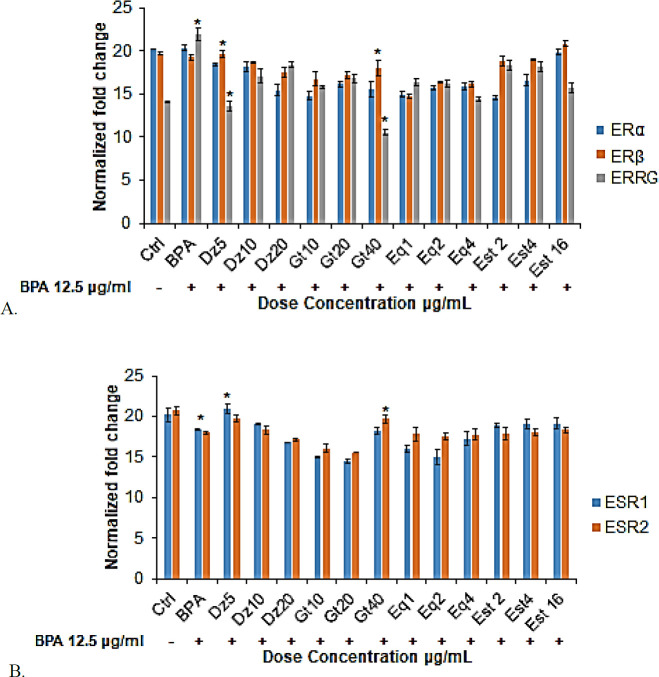
qRT-PCR analysis of oestrogen and oestrogen-related receptors in BPA-induced phytoestrogen-treated hFOB 1.19 cells under BPA exposure. Specific primer sequences: Table 1: were used for each gene A; oestrogen-related receptor genes: ERα, ERβ, ERRG, B; oestrogen receptors: ESR1, ESR2. All the genes expression in each group was normalized against HPRT1 and ACTB1 (reference genes). For BPA group, the cells were normalized again with Ctrl group and all the other treated groups were normalized with BPA group under normalized fold change. Data are expressed as mean±SEM triplicate independent biological samples. *P<0.05; is considered significant ERα: oestrogen receptor alpha, ERβ: oestrogen receptor beta, ESR1: oestrogen receptor 1, ESR2: oestrogen receptor 2, ERRG: oestrogen-related receptor gamma, BPA: Bisphenol A, Ctrl: Control

Since BPA decreased cell proliferation and differentiation associated genes, we also observed the expression of oestrogen and oestrogen-related receptors following 24 hr of incubation period. As observed in Figure 4A, B, we found that exposure with BPA alone had relatively similar expression of ERα and ERβ genes when compared to the Ctrl group (Figure. 4A). However, genes associated with oestrogen and oestrogen-related receptors like oestrogen receptor 1 (ESR1) and oestrogen receptor 2 (ESR2) were found to be significantly decreased (*P<*0.05) in BPA group (Figure 4B). A significant up-regulation (*P<*0.05) of ERRG was observed in BPA group compared to the Ctrl group. On the other hand, treatment with phytoestrogen caused increased expression of classical oestrogen related receptor genes including ERα and ERβ (Figure 4A). However, the non-classical ERRG was relatively decreased in (*P<*0.05) all the phytoestrogen and Est-treated groups compared to the BPA group (Figure 4A). When compared with BPA group of cells (Figure 4A), we found that in general, Dz5 and Gt40 groups maintained the bone health by significantly decreasing the expression of ERRG gene under BPA exposure. In addition, ESR1 and ESR2 were found to be well expressed following phytoestrogens treatment (Figure 4B). To be more specific, ESR1 showed significant (*P<*0.05) expression in Dz5 group, whereas Gt40 group showed more pronounced effect on ESR2 expression. Therefore, it is believed that phytoestrogens exert osteoprotective effect via increasing the expression of both ESR1 and ESR2. 

## Discussion

BPA is exposed to the industrial human populations constantly as it is easily found in environment in the form of plastic products, and factory or industrial wastes. BPA acts as a selective oestrogen receptor modulator and thus it is recognized as a xenoestrogen in general. Moreover, BPA does not show any structural homology with endogenous oestrogen E2 (19). However, it affects the ESR in similar or stronger way than E2 through non-classical oestrogen triggered pathways, which results in a larger ability to recruit co-activators (20). In general, BPA exhibits oestrogen antagonistic effects depending on both the ER subtype and the tissue involved (21). One of the most significant target organs of oestrogen is bone. In the bone cells, BPA significantly suppressed ALP activities, while E2 expression was found to be significantly increased (22). On the other hand, phytoestrogens exhibit the osteoprotective effect and was shown to improve bone loss by exerting the oestrogenic effect mediated through oestrogen receptors (23). Although osteogenic actions of BPA and phytoestrogens have been reported in several studies, the detailed mechanism of their effect towards oestrogen and its related receptors has not been elucidated.

Under BPA exposure, bone metabolism is disturbed as it inhibits the osteoblasts proliferation and differentiation (24). This study investigated the oestrogen-mediated pathway of BPA and phytoestrogens in hFOB 1.19 cells. We found that BPA has similar effect on ERα, but it relatively reduced the protein level of ERβ when compared to the Ctrl group. It is well known that both ERα and ERβ are expressed in osteoblasts and their precursor cells. They play a crucial role in bone remodelling in which ERα is responsible for bone development and maintenance processes (25), whereas ERβ prevents the stimulation of ERα in bone formation by regulating the activity of ERα (26). However, other researchers have reported that both ERα and ERβ act on the bone metabolism, osteogenic cytokines expression and osteoblast function (27). Moreover, it was shown that activation of ERβ was able to inhibit the decreasing osteoblast viability due to ERα depletion. Although BPA was shown to have similar effect like endogenous oestrogen, it affects only the ERα and not the ERβ. To gain further access, we analysed the effect of BPA on ERRG in hFOB 1.19 cells. The cells induced with BPA 12.5 μg/ml showed significant increase (*P<*0.05) in ERRG compared to Ctrl group. Previous data showed that BPA exerts anti-oestrogenic effect via non-classical oestrogen pathway (20). Our result is in agreement with previous study, which reported that BPA binds with high affinity to the ERRG, a nuclear receptor with unclear physiological ligand (3). Using an ERRG global knock-out mouse, Cordelli and Aubin (2014) found that ERRG does not seem to be required for bone development in mice, although the receptor acts in a runt related transcription factor 2 (RUNX2) -dependent manner (4). In an earlier study, Byung-Chul Jeong *et al*. (2009) found that inhibition of ERRG expression significantly increased bone morphogenetic protein 2 (BMP2)-induced osteoblast differentiation, osteocalcin expression, ALP activity and bone mineralization (5). Therefore, binding of BPA to ERRG receptor results in reducing the osteocalcin expression, and ALP activity and causes bone demineralisation. Our qRTPCR analysis also showed up-regulation of ERRG expression following 24 hr of BPA exposure. 

However, following treatment with phytoestrogens in the presence of BPA (12.5 μg/ml), both ERα and ERβ protein levels were relatively increased. Our study is in accordance with previous study in which researcher showed that genistein (Gt) induced ERα expression in MC3T3-E1 cells (28). They proved that mRNA expression of ERα was increased following Gt treatment. However, the study did not highlight the ERβ expression in Gt treatment. In the present study, we found that high concentration of Gt (40 μg/ml) insignificantly increased the ERα protein expression, and significant effect was observed with ERβ expression in hFOB 1.19 cells induced with BPA. The mRNA expression also showed up-regulation of both ERα and ERβ; however, the pronounced effect was observed in ERβ mRNA expression. In an earlier study, daidzein (Dz) was reported to enhance cell proliferation via ER-dependent MEK/ERK and phosphatidylinositol-3-kinase/ protein kinase B (PI3K/Akt) pathways in MG-63 cells (29). The effects of daidzein on cell apoptosis, ALP activity, and collagen content were mediated by both ERα and ERβ (30). Our study also showed that lower concentration of Dz (5 μg/ml) increased the ERα and ERβ protein levels in the presence of BPA. Similar results were found in mRNA expression of both ERα and ERβ following treatment with 5 μg/ml of Dz. Equol, a metabolite of Dz, inhibited the bone loss in ovariectomized mice (31) and promoted osteoblast proliferation and differentiation via activating the ER pathway (32), which further supported our results. Eq (4 μg/ml) showed increase in ERα and ERβ protein as well as mRNA expressions. However, the results were not significant when it compared to the other phytoestrogen groups. Simultaneously, we found that high concentration of Est (16 μg/ml) induced both ERα and ERβ expressions under BPA exposure. 

In contrast to the BPA alone group, all the treated groups (phytoestrogens and Est groups) showed significantly decreased ERRG protein and mRNA expressions under BPA exposure. It showed that phytoestrogens act on the osteoblasts by binding to the classical oestrogen receptors rather than the non-classical oestrogen receptor like ERRG. ESR1 and ESR2 mRNA expressions were upregulated in phytoestrogen-treated cells. So far, to our knowledge, little or no studies reported the molecular mechanism behind the effect of phytoestrogens towards BPA-induced bone loss. Our previous data also showed the increased cell viability, proliferation and differentiation of BPA-induced osteoblasts following treatment with different concentrations of phytoestrogens (17). From the present study, we underlined that phytoestrogens acts on BPA-induced osteoblasts as an ER agonist through activating the oestrogen and oestrogen-related receptors with significantly upregulating ERβ expression and downregulating the ERRG expression in hFOB 1.19 cells. Moreover, the direct effect of phytoestrogens on osteoblastogenesis was observed via upregulating the classical oestrogen receptors and downregulating the non-classical oestrogen receptor.

In regulating the bone remodelling, cytoskeletal organisation plays an important role. With cellular mechanical adaptation through cytoskeletal rearrangement of osteoblasts, there is an increased bone formation and function. In our study, the BPA group showed a smaller amount of dense actin filaments, which results in relatively deformed and less stiff osteoblasts. The stiffness of osteoblasts and the morphological appearance of cytoskeleton are influenced by oestrogen (33). Eventually, it is possible that the lower binding affinity of BPA to classical oestrogen receptors and higher binding affinity to non-classical oestrogen receptor caused anti-oestrogenic effect that induced mechanical changes to the osteoblasts and exerted the downstream effects. However, in the treated groups, Dz5, Gt40, Eq4 and Est (16 μg/ml) groups showed denser actin filaments in osteoblast cells. Previous data showed that Dz induced rapid changes in actin cytoskeleton in osteoblasts (34). However, in terms of the effect of phytoestrogens towards the density and filament composition, less data was found. We observed that the effect of phytoestrogens towards cytoskeleton arrangement of osteoblast-like cells is induced with BPA. Though, we did not quantify the relative density and stiffness of the actin cytoskeleton in the hFOB 1.19 cells.

The ERK/MAPK pathway plays an important role in osteoblast differentiation. Blocking or suppressing the MAPK pathway causes disturbance in the bone metabolism (35). We found out that the BPA group showed decreased MAPK1, MAPK3 and MAPK7 mRNA expressions compared to the Ctrl group. Similar finding was also observed in previous study in which BPA downregulated the differentiation signals, such as MAPK and ERK in MC3T3-E1 cells (24). Earlier study on mice observed that the ERK/MAPK signaling pathway involves in osteoblast differentiation (36). Increased MAPK signaling promotes differentiation of mesenchymal cells into osteoblasts (37). Blocking of MAPK supressed osteoblast-specific gene expression in mature osteoblasts cells (38). Therefore, it is believed that BPA inhibited the osteoblast differentiation by reducing the MAPK expression in hFOB 1.19 cells. However, in the treated groups, significantly increased MAPK3 gene expression was observed in Dz5, and Gt40 μg/ml groups. Surprisingly, the phytoestrogens-treated cells showed more pronounced effect on MAPK3 expression rather than Est groups. MAPK1 expression was downregulated in all the treated groups. Genistein was reported to stimulate the osteoblast differentiation through p38 MAPK-Cbfa1 (Core binding factor 1) pathway in mouse bone marrow-derived mesenchymal stem cells (BMSC) (39). We showed that low concentration of Dz (Dz= Dz5 ug/ml) and high concentration of Gt (Gt=Gt 40ug/ml) stimulated the cell differentiation via activation of MAPK3 expression in osteoblast-like cells under BPA exposure. Future study on detailed mechanism and the action of phytoestrogens on different MAPK expressions is mandatory.

We also analysed the mRNA expression of cell surface GPR30 in hFOB 1.19 cells. We found that the BPA group showed significant decrease in GPR30 or GPER1 expression. GPR30 is well-expressed in all bone cells such as osteoblasts, osteoclasts, osteocytes and chondrocytes (40). Several experimental studies showed the effect of GPR30 on bone health (41). GPR30 involves in proliferation and differentiation of the osteoblasts cells. From the present qRT-PCR result, it was shown that BPA caused decreased osteoblast proliferation and differentiation via down-regulation of GPR30 expression. Prior study suggested that GPR30 regulates the bone homeostasis and believed that this receptor is involved in endochondral bone formation (42). Moreover, it was reported that GPR30-mediated activation of adenylyl cyclase affects the activation of ERK/MAPK, which shows the link between GPR30 and ERK/MAPK pathway (43). Our data supported the findings of previous study that downregulated MAPK expression were found along with down-regulation of GPR30 in BPA group when compared to the Ctrl group. GPR30 is involved in early osteogenic differentiation, and silencing this gene in MC3T3-E1 cells caused significant reduction in cell growth (44). In the present study, both the phytoestrogens and Est-treated cells showed up-regulation of GPR30 expression in hFOB 1.19 cells under BPA exposure. The significant difference was observed in Dz5 and Gt40 μg/ml groups. These findings evidenced that phytoestrogens activated the GPR30 expression and induced cell proliferation and early differentiation in hFOB 1.19 cells under BPA exposure. The increased MAPK3 expression was also found in Dz5 and Gt40-treated groups (as mentioned above). In general, phytoestrogens rescued the BPA-induced osteoblast-like cells from reduced proliferation and differentiation via upregulating the MAPK3 and GPR30 expressions. 

Our study has few limitations. First, we did not detect the direct effects of phytoestrogens on osteoblasts. Second, hFOB 1.19 cells are different from primary osteoblasts as they are immortalized cells and proliferate rapidly. These cells can form mineralized nodules under special culture conditions. However, hFOB 1.19 cell lines are easily available, homogeneous and consistent *in vitro* model to study the human osteoblast cells (45). Additionally, the osteoblasts from human cancellous bone largely express ERβ than ERα (46, 47). The hFOB cells from human trabecular bone preserve the native regulatory pathways on direct treatment with oestrogen. Moreover, these types of commercial cells line are reported to express the ERα and ERβ genes (45, 48). The concentration of BPA used in the present study needs further investigations to determine the lethal concentration (LD_50_) in *in vivo* studies and later, maximum and minimum toxicity concentrations in the human clinical trials. Therefore, our findings in this study pave the way for further investigation to identify the direct effects of phytoestrogens and their oestrogenic effect on the primary osteoblasts. Study like this needs further confirmation for any firm conclusion to be drawn with regards to the effect of phytoestrogens in overcoming the adverse reaction of BPA on bone by activating the classical oestrogen receptors.

**Table 1:** List of genes and the primer sequence used in qRT-PCR for gene experssion analysis

Gene name	Primer sequence (5’ to 3’)
HPRT	Forward ATAAGCCAGACTTTGTTGG Reverse ATAGGACTCCAGATGTTTCC
ACTB	Forward GACGACATGGAFAAAATCTG Reverse ATGATCTGGGTCATCTTCTC
ESR1	Forward GGAGTGTACACATTTCTGTC Reverse CAAAGTGTCTGTGATCTTGTC
ESR2	Forward AAATCTTTGACATGCTCCTG Reverse AGGGTACATACTGGAATTGAG
ERAlpha	Forward CATCCCAGGCTTCTCATC Reverse ACTAAGTCCTCAGCGAAG
ERBeta	Forward GTGGAAGAGAAATGAGCTTG Reverse AAACTTTTATTCACCAGCCC
ESRRG	Forward GTGATGTGTACCATACTGTG Reverse TTAGCAGTCAAAAGTGGAAG
GPER	Forward AGGTACCCAGAGAGTGAG Reverse AGTGGGAAGAACAGATGC
MAPK1	Forward GAAGCATTATCTTGACCAGC Reverse TCCATGGCACCTTATTTTTG
MAPK3	Forward TTCGAACATCAGACCTACTG Reverse TAGACATCTCTCATGGCTTC
MAPK7	Forward AGCACTTTAAACACGACAAC Reverse TAGACAGATTTGAATTCGCC

Dz: Daidzein, Gt: Genistein, Eq: Equol, ERα: oestrogen receptor alpha, ERβ: oestrogen receptor beta, ERRG: oestrogen-related receptor gamma, BPA: Bisphenol A

Dz: Daidzein, Gt: Genistein, Eq: Equol, Est: 17β-oestradiol, BPA: Bisphenol A, Ctrl: Control

BPA: Bisphenol A

## Conclusion

We investigated that BPA reduced osteoblast proliferation and differentiation due to its higher binding affinity to the non-classical oestrogen receptor like ERRG receptor. In contrast, different types of phytoestrogens increased the hFOB 1.19 cells differentiation via activating the ERα and ERβ receptors and supressing the ERRG receptor expression. The oestrogenic activity of phytoestrogens was triggered by the increased density of actin filaments in osteoblast-like cells under BPA exposure. Specifically, low concentration of daidzein and high concentration of genistein is required for augmenting the osteoblast differentiation via MAPK pathway and GPR30 receptor expression. The osteoprotective effect of Eq may in part enlighten the lack of benefits of Eq in bone research. These interesting findings also explained the less positive effect of Eq compared to Dz and Gt in maintaining the bone health. Therefore, it is crucial to study the molecular mechanism of different types of phytoestrogens to explore their therapeutic roles in the *in vitro* and *in vivo* studies, as well as in clinical trials. Considering that oestrogen and oestrogen-related receptors to be the most preferred molecular targets, phytoestrogens possess multiple positive effects that make it a potential alternative supplement in overcoming the bone damage due to BPA exposure.
